# Are monocytes a preferable option to develop myeloid cell-based therapies for solid tumors?

**DOI:** 10.1186/s13046-025-03359-x

**Published:** 2025-03-15

**Authors:** Daisy Bhatia, Riccardo Dolcetti, Roberta Mazzieri

**Affiliations:** 1 Swiss Federal Institute of Technology, Lausanne, Switzerland; 2 Peter MacCallum Cancer Centre, Melbourne, VIC 3000 Australia; 3 Sir Peter MacCallum Department of Oncology, The University of Melbourne, Melbourne, VIC 3000 Australia; 4 Department of Microbiology and Immunology, The University of Melbourne, Melbourne, VIC 3000 Australia; 5 Faculty of Medicine, University of Queensland, Brisbane, QLD 4102 Australia

**Keywords:** Immunotherapy, Adoptive cell therapy, Chimeric antigen receptors, Macrophages, Monocytes

## Abstract

In the last two decades, novel and promising cell-based therapies have populated the treatment landscape for haematological tumors. However, commonly exploited T and NK cell-based therapies show limited applicability to solid tumors. This is mainly given by the impaired tumor trafficking capability and limited effector activity of these cells within a highly immunosuppressive tumor microenvironment. Myeloid cells spontaneously home to tumors and can thus be reprogrammed and/or engineered to directly attack tumor cells or locally and selectively deliver therapeutically relevant payloads that may improve the efficacy of immunotherapy against difficult-to-access solid tumors. In the context of myeloid cell-based therapies, adoptive transfer of monocytes has often been overshadowed by infusion of differentiated macrophages or hematopoietic stem cell transplantation despite their promising therapeutic potential. Here, we summarize the recent improvements and benefits of using monocytes for the treatment of solid tumors, their current clinical applications and the challenges of their use as well as some possible strategies to overcome them.

## Introduction

In recent years, T and NK cell-based therapies have revolutionized cancer treatment, with promising clinical success stories in patients with haematologic tumors. However, a broad applicability of these cell-based therapies to solid tumors is still limited by some key practical challenges, significant costs, manufacture times and patient-to-patient variability [1–3]. Moreover, the inhibited recruitment, infiltration and persistence of effector T and NK cells dramatically reduce the therapeutic efficacy of cellular immunotherapies in solid tumors. Most of these obstacles are mediated by tumor infiltrating myeloid cells educated by the tumor to play immunosuppressive roles that actively promote malignant progression and immune evasion [4, 5]. However, taking advantage of the spontaneous ability of myeloid cells to reach the tumor microenvironment (TME), several attempts have been made to either reprogram these cells and/or use them for drug delivery to solid tumors. Myeloid cells, initially only considered as therapeutic targets to reverse local immunosuppression, are now demonstrating several advantages over T and NK cell-based cellular therapies, including the above-mentioned tumor homing properties for efficient and targeted drug delivery, their long-lasting persistence within a tumor, as well as their fundamental role in promoting and directing the crosstalk between innate and adaptive immunity if appropriately reprogrammed. This can be achieved by either adoptively transferring mature macrophages that have been ex vivo genetically engineered and/or pharmacologically modulated, or by transplanting hematopoietic stem cells (HSCs) specifically engineered in their myeloid progenies. More recently, monocytes are emerging as promising alternatives to mature macrophages and HSCs due to their enhanced availability, easy isolation, minimal ex vivo culture requirements and enhanced tumor homing proprieties.

Here, we summarize the main challenges associate with T, NK and myeloid cell-based therapies and highlight the most recent advancements in the use of monocytes as valid and clinically applicable alternative.

### T & NK cell-based cellular therapies

The most advanced and successful adoptive cell therapies (ACTs) employ different strategies to enhance the activity of effector T lymphocytes and promote anti-tumor immune responses. These include generation of chimeric antigen receptor (CAR) T-cells, T-cell receptor (TCR) gene-modified T cells, and isolation and expansion of tumor-infiltrating lymphocytes (TILs), each presenting with specific advantages and disadvantages (extensively reviewed in Albarran et al., Frontiers in Immunology 2024) [6].

**Fig. 1:**
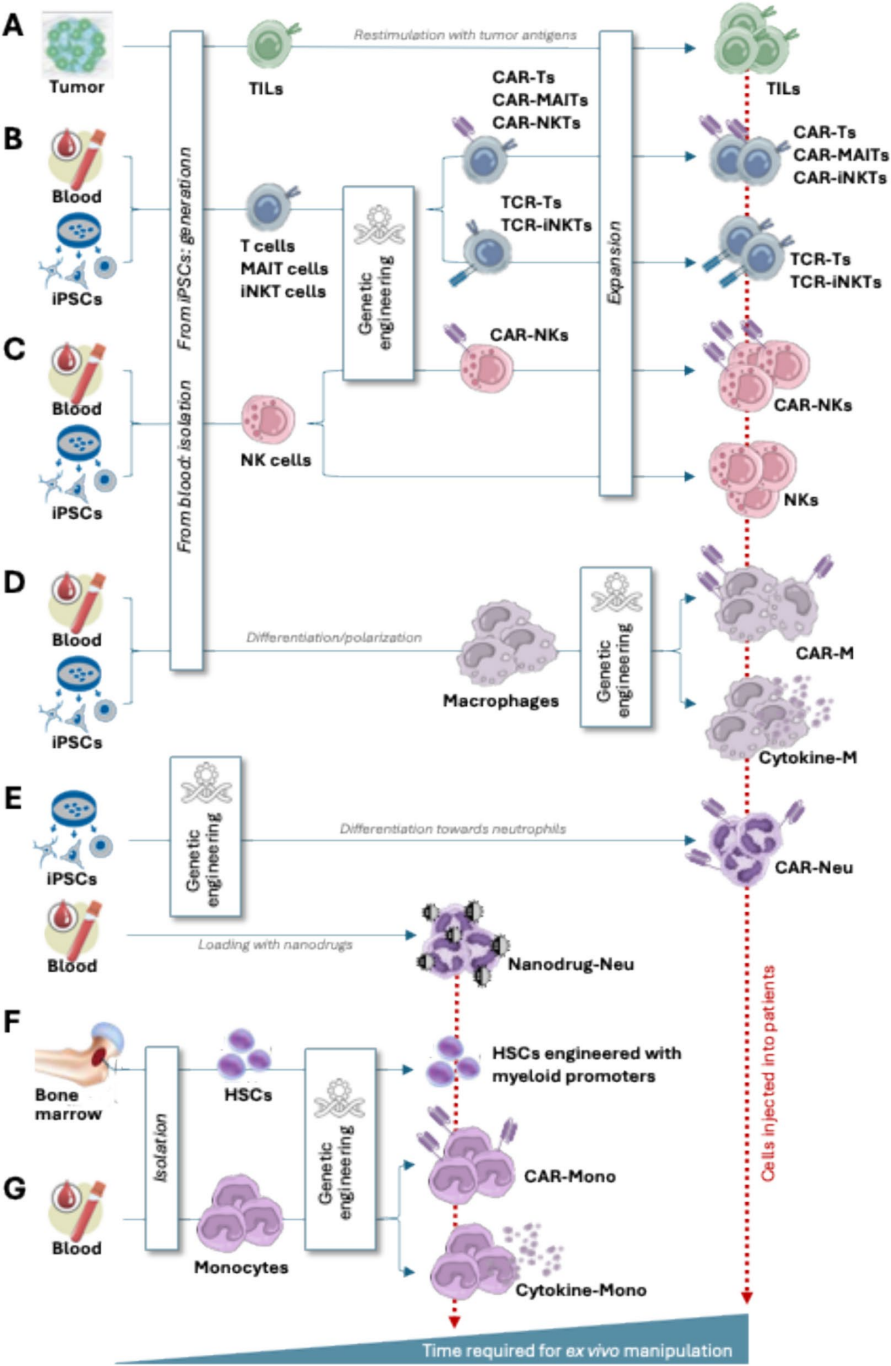
Cell based therapies for the treatment of solid tumors: sources of immune cells and their ex vivo manipulation. **A** Tumor infiltrating lymphocytes can be isolated from available tumor tissue, re-stimulated with tumor antigens if known, and expanded before injection into the patient. **B,C** Autologous T, MAIT, iNKT (B) and NK (C) cells can be isolated from patient’s blood, genetically engineered ex vivo and/or expanded before reinjection. **D** Macrophages can be differentiated starting from blood-derived monocytes or iPSC-derived myeloid progenitors, engineered, and reinjected into the patient. **E** Neutrophils can be differentiated starting from iPSC-derived myeloid progenitors, engineered, and infused into the patient or isolated in large amounts from the patient blood, loaded with nanodrugs and immediately reinjected into the patient. **F** Autologous bone marrow derived HSCs can be isolated and engineered in few days and reinjected into preconditioned patients. **G** Monocytes can be easily isolated in large amounts from the patient’s blood, engineered in few hours and immediately infused into the patient

In CAR-T cells (Fig. 1B), the extracellular domain of a chimeric antigen receptor (CAR) allows the recognition of selected tumor antigens. However, it can only recognize extracellular antigens. On the contrary, engineered TCR-T cells (Fig. 1B) can recognize a wider repertoire of tumor antigens including intracellular antigens presented by specific MHC molecules, which, however, might benefit a restricted number of patients depending on their human leukocyte antigen (HLA) haplotype. With both types of cell therapies, the antigens must be homogeneously expressed by tumor cells, which is almost never the case in solid tumors, especially as they progress to metastatic disease, typically characterized by genomic instability and antigenic heterogeneity, both contributing to antigen escape, a well-known immune evasive mechanism in this setting. Moreover, in solid tumors, these therapies often target antigens that are not tumor specific, thus increasing the probability of on-target off-tumor (OTOT) toxicity. TIL therapy (Fig. 1A) relies on the isolation and expansion of tumor infiltrating T cells. Because TILs are reactive against multiple tumor antigens, they have the great advantage of reducing the probability of both antigen escape and OTOT, making them especially useful for the targeting of changing and heterogeneous solid cancers. However, TILs isolation requires tumor tissue availability as well as expensive and logistically complex process for their expansion [7, 8].

**Fig. 2:**
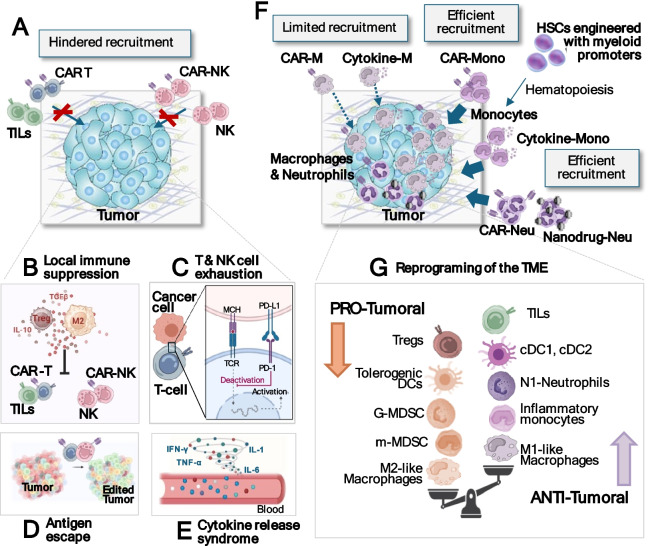
Cell-based therapies for the treatment of solid tumors: tumor recruitment, persistence, and local and systemic effects upon cell injection. **A** Injected T and NK cells are poorly recruited to the tumor sites due to lack of recruitment factors, abnormal blood vessels or extracellular matrix (ECM), and down-regulation of extravasation mediators. **B** T and NK cells that manage to infiltrate the tumor are rendered dysfunctional by many factors, such as interleukin (IL)−10 and transforming growth factor beta (TGF-β) secreted by immunosuppressive regulatory T (Treg) cells, M2 macrophages and myeloid-derived suppressor cells (MDSCs). **C** Activation of inhibitory immune checkpoint pathways, such as the programmed cell death protein 1 (PD-1) / programmed cell death protein 1-ligand (PD-L1) axes, also contributes to a dysfunctional phenotype of tumor infiltrating T and NK cells. **D** Injected T and NK cells upon activation and proliferation can trigger systemic inflammation which leads to the release of pro-inflammatory factors, such as cytokines, sometimes resulting in systemic toxicity such as cytokine release syndrome and neurotoxicity. **E** Tumor heterogeneity, immune editing and reduced antigen presentation prevent recognition and killing from injected T and NK cells. **F** Injected monocytes, monocytes derived from injected HSCs and neutrophils are more efficiently recruited to solid tumors than T or NK cells and macrophages. **G** Macrophages or monocyte-derived macrophages engineered to deliver pro-inflammatory cytokines, can reverse local immunosuppression and promote anti-tumor immune responses including increased recruitment of effector innate and adaptive immune cells

Another important challenge is the applicability and efficacy of T cell-based therapies against solid tumors, which are strongly limited by their reduced accessibility compared to haematological cancers [1, 9]. Indeed, infusion of T cells in patients with solid tumors usually shows inefficient recruitment, extravasation and tumor infiltration [1, 9] (Fig. 2A). Trafficking to the tumor site is also often hindered by a highly immunosuppressive stroma and abnormal tumor vasculature [3, 9]. Furthermore, even upon tumor infiltration, T cells’ effector functions may be severely inhibited by tumor-induced immunosuppression (Fig. 2B), chronic exposure to tumor antigens and consequent exhaustion (Fig. 2C) as well as inevitable immune editing and loss of targetable antigens (Fig. 2D) [3, 9, 10].

Finally, up to 30% of patients treated with T cell-based therapies develop significant immune-mediated toxicities such as the cytokine release syndrome (CRS) and the immune effector cell-associated neurotoxicity syndrome (ICANS) (Fig. 2E), which require specific and expensive treatments in specialized centers that are experienced in managing the common toxicities of CRS and ICANS and by the financial and health burden that this creates [11].

Another major limitation in the applicability of T cell-based therapies is the time-consuming manufacturing often associated with low efficacy and patient-to-patient variable outcomes. The use of T cells derived from induced pluripotent stem cells (iPSCs) may overcome some of these limitations and provide an off-the-shelf source of engineered T cells. Indeed, iPSCs can grow infinitely, are easy to genetically manipulate, and provide safe stable clonal lines. More needs to be done, however, to develop engineering as well as differentiation protocols that are better compliant with good manufacturing practice (GMP) standards, scalability, and safety measures [12, 13].

Like T cells, NK cells (Fig. 1C) have also been extensively explored for the development of cell-based immunotherapies including autologous, allogeneic, progenitor-derived or iPSC-derived NK cells which are either in vitro pre-activated or genetically engineered (extensively reviewed in Vivier, Nature 2024) [14]. As for T cells, strategies to enhance trafficking to solid tumors and promote persistence as active effector cells are required to improve NK cell-based immunotherapies.

Recently, Mucosal-associated invariant T (MAIT) cells (Fig. 1B) have also been considered for programming with chimeric antigen receptors (CARs). Despite the exact role of MAIT cells in cancer remains uncertain, the use of CAR-MAIT cells might overcome some of the limitations seen with CAR Ts. They release lower levels of inflammatory cytokines, thus reducing risks of CRS. Their restricted TCR prevents them from inducing graft-versus-host disease (GvHD), making them ideal candidates for the development of allogeneic off-the-shelf cell therapies in cancer. Finally, their abundance in the TME and their natural ability to infiltrate chronically inflamed tissues strongly support their use in targeting solid tumors. However, more preclinical in vivo work is required to prove their therapeutical potential [15–18]. Of note, to overcome suboptimal ex vivo expansion efficiency of MAITs, iPSC-derived MAITs were also tested for their anti-tumor proprieties in murine cancer models [19, 20].

Similarly, when compared with T cells, invariant natural killer T (iNKT) cells (Fig. 1B) show superior tumor infiltrating abilities, and, importantly, do not promote GvHD, allowing the design of of-the-shelf therapies. However, as for any type of allogenic cell, graft rejection is expected, thus requiring further genetic editing to knocking out or knocking down expression of HLA class I and II molecules, or include expression of “don’t-eat-me” signals. Several strategies have been explored to redirect iNKT cell against cancer cells, including CD1d-antibody fusion proteins, CARs, and tumor-specific TCRs. The extremely low frequency of iNKT cells in the human blood is the main limitation to their clinical application and prompted the development of novel protocols where genetic engineering of HSCs or iPSCs is combined with differentiation protocols for large-scale productions of CAR- or TCR-NKT cells [21].

There are several ongoing clinical trials testing T/NK based cell therapies [22]. However, given the above-described limitations in their applicability in solid tumors, several efforts are being made to overcome current challenges including developing novel therapeutic combination strategies to enhance specificity, infiltration, and efficacy of T/NK cell-based treatment and to modulate the inhibitory conditions within the TME. At the same time, alternative immune effector cells that can be engineered to develop antitumor cellular immunotherapy have been explored, especially those within the myeloid compartment.

### Myeloid cell-based therapies

Myeloid cells comprise macrophages, dendritic cells, monocytes, and granulocytes and are the major immune cell populations recruited to the TME [23] as a result of the release of chemokines produced by both cancer and stomal cells, including CCL2, VEGF, Angiopoietin-2, CXCL12, CSF-1 [24–28]. Upon tumor infiltration, the heterogeneous, dynamic, and constantly evolving interactions of myeloid cells with cancer cells and the stroma promote their polarization towards a spectrum of different functional stages ranging from pro- to anti-tumoral [29, 30]. The relative balance between these opposite states of activation determines the level of local immune suppression. In addition, factors released by tumors can promote the pathological differentiation of myeloid cell progenitors in the bone marrow that can affect their future polarization within the tumor microenvironment. Pathologically activated myeloid cells can have distinct transcriptional profiles resulting in specific functional activities but limited phenotypic markers for a specific classification into subpopulations [5]. However, the natural tumor homing capability of myeloid cells offers attractive cell engineering opportunities that can be exploited for the development of cell-based therapies for solid tumors.

The myeloid lineage has been mainly exploited therapeutically by either manipulating the early lineage progenitors in HSCs in transplant settings (Fig. 1F), or by engineering terminally differentiated macrophages (Fig. 1D and Table 1) or neutrophils (Fig. 1E). Only recently, monocytes have also been considered for cell-based therapies (Fig. 1G and Table 1).

**Table 1:**
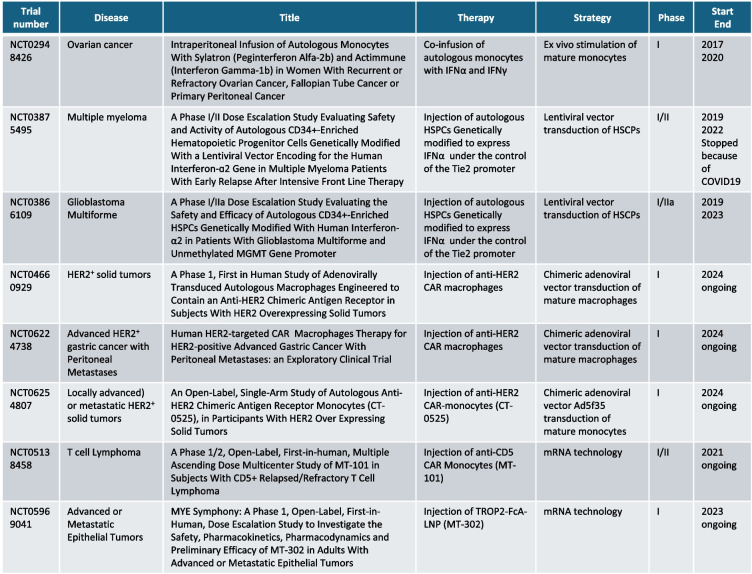
Current clinical trials on myeloid cell-based therapies for cancer treatment. Source: clinicaltrials.gov/

### Haematopoietic Stem Cell (HSCs) transplantation

When compared with other stem cell therapies, HSC transplantation has the advantage of relatively easy access to large amounts of stem cells and the availability of well-characterized and clinically tested procedures for their therapeutic use in several contexts, including Wiskott-Aldrich syndrome, X-linked severe combined immunodeficiency, β-thalassemia, and adrenoleukodystrophy, a severe demyelinating brain disease [31–35]. However, the wide distribution of HSC progeny in different tissues leads to systemic rather than localized delivery of the therapy and therefore potential toxicity.

In transplantation settings, myeloid-specific gene regulatory regions have been exploited to deliver therapeutic biomolecules within the tumor (Fig. 1F). By transducing HSCs with specifically engineered viral packages, the derived monocytes can be leveraged as tumor-homing cellular vehicles. Moreover, the use of promoters typically upregulated after monocytes enter the tumor and differentiate into tumor-associated macrophages (TAMs) can allow local targeted delivery of the cargos (Fig. 2F). In particular, the Tie2 promoter and the matrix metalloproteinase-14 transcriptional control elements were successfully exploited to deliver interferon (IFN)-α [36–40] and TNF-related apoptosis-inducing ligand or a TGF-β blocking protein, respectively [39, 40]. The matrix metalloproteinase-14 (MMP14) promoter was selected as the strongest promoter able to limit gene expression to myeloid cells infiltrating brain metastasis or glioblastoma (GBM) in mice [39, 40]. Using this strategy, the tumor targeted delivery of the soluble TGF-ß receptor II significantly improved the outcome of radiotherapy in a model of murine GBM. Of note, increased survival was associated with long-term memory protection against tumor rechallenge [40].

In the context of solid tumors, to date, a phase I/IIa clinical trial has been completed on the use of HSCs engineered to express IFN-α under the control of the Tie2 promoter (Temferon) in their tumor homing and Tie2^+^ macrophage progeny for the treatment of GBM (NCT03866109) [41]. Tie2 is indeed expressed at very low levels in the periphery and at significantly higher levels within the tumor [42]. Rapid engraftment of gene modified progenitors was observed in all patients, as given by the presence of vector genomes in the DNA. In 2 out of 2 fresh tumor samples analyzed, 3% to 5% of gene-marked tumor infiltrating CD45^+^ cells were detected indicating homing of the engineered macrophages to the TME. Of note, overall Interferon Stimulated Genes (ISGs) expression was increased when first and second surgery tumor tissues were compared thus supporting local delivery of biologically active IFN-α [43]. Moreover, very low IFN-α concentrations were detected in plasma and cerebrospinal fluid (CSF), indicating tight regulation of transgene expression. As shown by single cell RNA sequencing, the tumor targeted delivery of IFN-α resulted in the reprogramming of the myeloid compartment towards pro-inflammatory phenotypes and increased recruitment of effector T cells. Despite all treated patients experienced progressive disease, those undergoing a second-line treatment (57%) presented a 28% interim survival rate at 2-years, which is higher than the 15% reported in the literature. Most severe adverse effects were associated with conditioning chemotherapy (e.g. infections) or disease progression (e.g. seizures). Altogether, the trial demonstrated the safety of the treatment and its potential to counteract disease progression and improve survival of GBM patients [44]. In 2023, the European Commission has granted Orphan Drug Designation to Temferon™ for the treatment of glioma and a phase II clinical trial will start soon.

Engraftment of stably engineered HSCs has the advantage of providing a continuous source of IFN-α-producing macrophages ready to deliver their cargo to tumors. However, chronic exposure to certain biomolecules, such as type I IFNs, can be deleterious and could promote tumor progression as it correlates with higher T cell exhaustion, evasive resistance and poor patient survival [45]. To counteract these limitations, a second-generation gene delivery platform is being investigated that allows on/off switching of cytokine secretion at the disease site. The method was applied to both IFN-α and IL-12 delivery and showed synergistic efficacy with checkpoint blockade or CAR-T cell therapy in mouse tumor models [46].

HSC transplantation can only be performed in well-defined clinical settings and is not applicable to all patients. Indeed, this strategy is burdened by several limitations including: insufficient numbers of HSCs obtainable for effective cell therapy, and possible side effects deriving from the profound bone marrow suppression, which requires time for recovery and can be associated with opportunistic infections, veno-occlusive liver disease, interstitial pneumonia, graft failure, and infertility.

## Neutrophil-based cell therapies

Neutrophils are the most abundant circulating immune cells and efficiently home to tumors due to their strong inflammatory responsiveness (Fig. 2F). Several strategies have been designed to exploit neutrophils or neutrophil derivates (membranes and extracellular vesicles) as drug carries to achieve tumor-targeting delivery by intrinsic or induced tumor-associated inflammation (extensively reviewed in Zang, Advanced Materials, 2024) [47]. When considering neutrophils as living cell drug delivery systems (Nanodrug-Neu, Fig. 1E), extensive work has been performed to design nanomaterials to load different types of drugs on autologous neutrophils isolated from patients’ blood (ex vivo loading), or directly in vivo on circulating neutrophils (a hitchhiking strategy). Indeed, neutrophil-mediated transport was shown to be crucial for delivering nanoparticles to tumors as neutrophils prevent short-circulating nanoparticles from being quickly cleared and facilitates their extravasation into the TME [48, 49]. Examples include neutrophil-mediated delivery of chemotherapeutic drugs, acoustic sensitizers, photosensitizers, as well as targeted cancer theranostics [47]. The ability of neutrophils to cross physical barriers such as the brain blood barrier or the bone-marrow barrier, makes them ideal delivery systems to reach difficult to treat tumors such as recurring brain or bone primary and metastatic cancers [50, 51]. Most of the strategies described above directly load neutrophils in vivo (hitchhiking strategies) due to their innate ability to uptake, thus eliminating expensive and time-consuming isolation steps. However, in vivo clearance of nanomaterials by the mononuclear phagocyte system limits its applicability. Both in vivo and in vitro loading of neutrophils, require a careful design of the nanomaterials aiming for high binding affinity and specificity to avoid off-target side effects. All studies so far were performed in animal models and, therefore, specific clinical trials should be designed to test long-term safety, tolerance and therapeutic potential of neutrophil-based living cell drug delivery systems.

Neutrophils are also short-lived and resistant to genetic alterations, for this reason they have not been manipulated with CARs as mature cells. However, Chang et al., successfully engineered iPSCs and differentiated them into neutrophils (CAR-Neu, Fig. 1E) efficiently targeting and killing tumor cells both in vitro and in vivo [52]. Of note, this would allow the generation of large quantities of CAR-Neu that can be further loaded with specific nanodrugs before injection into the patient [53].

### Macrophage-based cell therapies

Macrophages as adoptively transferred vehicles for cell therapy are also currently under active investigation (Fig. 1D). The tumor homing and phagocytic capabilities of macrophages can be harnessed using different modulatory and/or engineering strategies, to achieve either cytotoxic effector functions, or local delivery of therapeutic proteins and immunomodulatory agents [54, 55] able to reprogram the adoptively transferred macrophages and/or the TME (Fig. 2F & G). These approaches include the expression of cytokines like IL-12, or IL-21 [56–58], the secretion of cytotoxic agents [59], or the inhibition of immunosuppressive genes like SIRPα with Clustered Regularly Interspaced Short Palindromic Repeats-CRISPR-associated 9 (CRISPR-Cas9) [60]. As in the case of neutrophils, macrophages can be loaded with nanodrugs to be delivered to tumors (reviewed in Yang, Cell Death and Disease, 2024) [55]. Availability of peripheral blood monocytes facilitates the implementation of macrophage-based therapies in the clinic. Moreover, efficient protocols for their differentiation from iPSCs have also been developed and can provide an unlimited source of macrophages [61, 62].

#### Chimeric Antigen Receptor Macrophages (CAR-M)

CAR-M represent the most advanced and promising engineered macrophage therapy for solid tumors [62–65]. They can mediate tumor cell phagocytosis in a CAR-dependent and CAR-independent manner and they can also present antigens, provide co-stimulatory signals, and secrete cytokines, which recruit and activate other immune cells to participate in anti-tumor responses. Moreover, in contrast to T cells, severe CRS rarely occurs with CAR-Ms, probably due to their limited expansion potential and transient persistence in peripheral blood [66]. The CAR constructs exploited in CAR-M share the main structural components used for CAR-T engineering with intracellular activation signaling regions, transmembrane regions and extracellular signaling domains to recognize tumor antigens. The various generations of CAR-M developed so far were recently extensively reviewed [64, 67, 68].

First-generation CAR-Ms were designed to promote phagocytosis of cells expressing specific targets. This was accomplished by designing Chimeric Antigen Receptors for Phagocytosis (CAR-Ps) where standard extracellular and transmembrane CAR domains were combined with cytoplasmic domains capable of promoting phagocytosis such as Megf10 (multiple EGF-like-domains protein 10), a member of the multiple epidermal growth factor-like domains protein family, or FcRγ (Fc receptor γ-chain) an immunoreceptor tyrosine-based activation motif (ITAM)-containing Fc receptor [69]. Moreover, the internalization of large targets was promoted by introducing also the Phosphoinositide 3-kinase (PI3K) signaling motifs, known for their ability to increase the frequency of whole-cell engulfment by threefold [70]. In 2020, the intracellular domain CD3ζ was also explored to generate anti-HER2 CAR-Ms [71]. The tandem SH2 in kinase Syk in macrophage binds to CD3ζ and triggers the phagocytosis in macrophages.

Second-generation CAR-Ms were aiming at improving antigen presentation and T cell activation ability by inducing and maintaining the pro-inflammatory phenotype of CAR-Ms while also massively expanding them to achieve optimal quantities for infusion [72]. Towards this goal, transduction with adenoviral vectors such as Ad5f35 was shown to promote the induction of a pro-inflammatory, anti-tumor phenotype that could be sustained for at least 40 days [71]. Enhanced expansion (> 50-fold) and persistence (about 30 days) of CAR-Ms was achieved by using induced pluripotent stem cells (iPSCs) to generate CAR-iMac [61]. Recently, CAR-iMac was engineered with a toll-like receptor 4 intracellular toll/IL-1R domain-containing CARs, which resulted in enhanced antigen-dependent polarization towards a pro-inflammatory phenotype in a nuclear factor kappa B-dependent manner [62]. Moreover, exploitation of nanobiotechnology and in vivo reprogramming approaches improved the anti-tumor efficacy of third-generation CAR-Ms [72]. Macrophage-targeting nanocarriers were successfully used to transfer plasmid DNA encoding CAR-interferon-γ into macrophages in vivo, which acquired a pro-inflammatory phenotype coupled with enhanced phagocytosis and anti-tumor immunomodulation ability [73]. However, the non-specific and variable uptake of these nanocarriers at the tumor site hampered the global therapeutic efficacy of this approach. This limitation was recently overcome by using a synthetic universal DNA nanocarrier functionalized with RP-182 peptides in the shell of nanoparticles able to specifically target macrophages [74]. Indeed, RP-182 peptides have been previously shown to bind and activate CD206 resulting in an efficient reprogramming of TAMs to acquire an anti-tumor phenotype. Treating with RP-182 peptides significantly inhibited tumor growth and extended survival in syngeneic and autochthonous murine cancer models, also showing the ability to enhance the efficacy of chemotherapy or immune checkpoint immunotherapy [75]. When RP-182-mediated targeting was incorporated into a human epidermal growth factor receptor 2 (HER2) CAR gene-laden DNA nanocomplex for in vivo generation of CAR-Ms, therapeutic efficacy was demonstrated in a preclinical model of brainstem glioma [74], indicating that CAR-M-based therapy may represent a promising avenue for these poorly amenable tumors. One of the main limitations in CAR-T therapy for solid tumors is the poor T cell recruitment due to lack of recruiting signals and/or the compact nature of the extracellular matrix. To overcome this limitation, the internal signaling domain of CD147 was introduced. When activated, this domain stimulated the production of matrix metalloproteinase-9 (MMP9), thus attenuating the deposition of tumor collagen and facilitating T cell infiltration. CAR-HER-2-CD147 specifically activates MMP expression in CAR-M within the TME upon recognition of the tumor antigen HER-2 [76]. While the antigen-binding domain of most CAR-Ms exploits the variable regions of monoclonal antibody heavy and light chains recognizing tumor antigens, natural receptor-ligand pairs were also successfully developed. CCL19, the ligand of CCR7, was selected to target CCR7-positive tumor cells, an immunosuppressive and highly invasive cell population. CCR7-targeting CAR-M were shown to significantly inhibit the growth of subcutaneous tumors induced by the 4T1 mouse triple negative breast cancer cell line [77].

To date, two clinical trials are ongoing to assess safety and efficacy of HER2-targeting CAR-M therapies in HER2-overexpressing tumors (NCT04660929) and in advanced HER2 + gastric cancer with peritoneal metastases (NCT06224738). Results from the NCT04660929 trial were recently reported [78] showing an overall safe profile with no dose-limiting toxicity, no severe CRS or ICANS. Moreover, 44% of tumors (4 out of 9 HER2 3 + tumors) achieved stable disease 8 weeks after treatment. These findings support safety, tolerability and manufacturing feasibility of HER2-targeting CAR-M therapies.

Although genetically engineered macrophages are showing promising results, a broader clinical application of this therapeutic approach is still hampered by some critical limitations. A key challenge is the required ex vivo differentiation step of autologous macrophages from circulating monocytes, which implicates considerable cell loss as well as a lengthy, expensive and difficult upscaling process. This limitation could be partially overcome using iPSC-derived macrophages. Macrophages do not induce GvHD, therefore the generation of iPSC-derived allogenic macrophages can be considered. The highly plastic properties of these cells make them susceptible to phenotypic and functional changes hard to control both in vitro during manufacturing and in vivo upon entering the tumor microenvironment. Fundamental will be the development of further engineering and/or the combination with other therapies to promote the acquisition of an anti-tumor phenotype by the therapeutic macrophages. Another challenge associated with the use of CAR-M concerns the reduced proliferation ability of macrophages after injection, resulting in a limited amount of circulating macrophages. However, there is evidence suggesting that, upon entering the TME, (TAMs are trained to proliferate by the tumor. In mouse models, TAMs in breast cancer tissues have higher proliferative capacity than macrophages in normal mammary tissue [79]. Therefore, the actual and long-term persistence of therapeutic macrophages in vivo within the tumor remains to be investigated. Importantly, the trafficking potential of injected macrophages is also suboptimal as compared to other immune cells, particularly monocytes and neutrophils (see below), as most of them stay in the liver [80]. Macrophage-based cell therapies also pose several safety concerns. The ability of these cells to penetrate in all organs and accumulate in the liver may lead to unexpected off-target toxicity. These key limitations provide the rationale for a possible shift towards monocyte-based therapies, which will be discussed in detail in the next paragraph.

### Monocyte-based cell therapies: advantages and current therapeutic applications

Using monocytes instead of macrophages or hematopoietic progenitors can offer several advantages and may potentially overcome some of the current challenges limiting the applicability and efficacy of myeloid-based cell therapies (Fig. 1G). Monocytes can be easily isolated from peripheral blood through leukapheresis, which allows for the collection of larger numbers of monocytes from a relatively small blood sample, making the process more straightforward and scalable for manufacturing. Monocytes also represent a relatively more homogeneous starting population than macrophages, making it easier to manipulate their differentiation or engineering process ex vivo [81]. This allows for a more controlled and standardized production process characterized by consistency and reproducibility. In addition, monocytes require reduced ex vivo growth and differentiation times as compared to macrophages, thus minimizing cell loss while retaining functional and therapeutic potential. This also decreases the risk of contamination and lowers production times and costs. Importantly, monocytes have superior trafficking abilities as compared to macrophages (Fig. 2F). Indeed, monocytes are constantly circulating in the bloodstream, which allows them to quickly migrate to sites of inflammation or tumors, whereas macrophages are often resident cells in tissues and may not migrate as rapidly [82]. Monocytes also respond more vigorously to attracting chemokines, particularly CCL2, thereby migrating more efficiently than macrophages [83]. Monocytes show a different profile of adhesion molecules and integrins than macrophages, which influences their ability to adhere to endothelium and extravasate into tissues [84]. Direct comparison experiments in in vivo models such as injury or infection demonstrated that monocytes can be recruited more rapidly than macrophages to inflammatory sites [85]. Furthermore, monocytes have a higher intrinsic proliferative potential, especially in response to inflammatory signals, than macrophages, which are more specialized and terminally differentiated cells. Although monocytes are considered to cease proliferation once released into the bloodstream, some studies have shown that certain subpopulations of monocytes retain the ability to proliferate under specific conditions [86, 87]. Taken together, the possibility that adoptively transferred monocytes could show a certain degree of expansion after reaching the tumor bed and differentiating into TAMs [79] is intriguing and, if convincingly demonstrated, it could further support the superiority of these cells over macrophages as cell therapy vehicles.

**Fig. 3:**
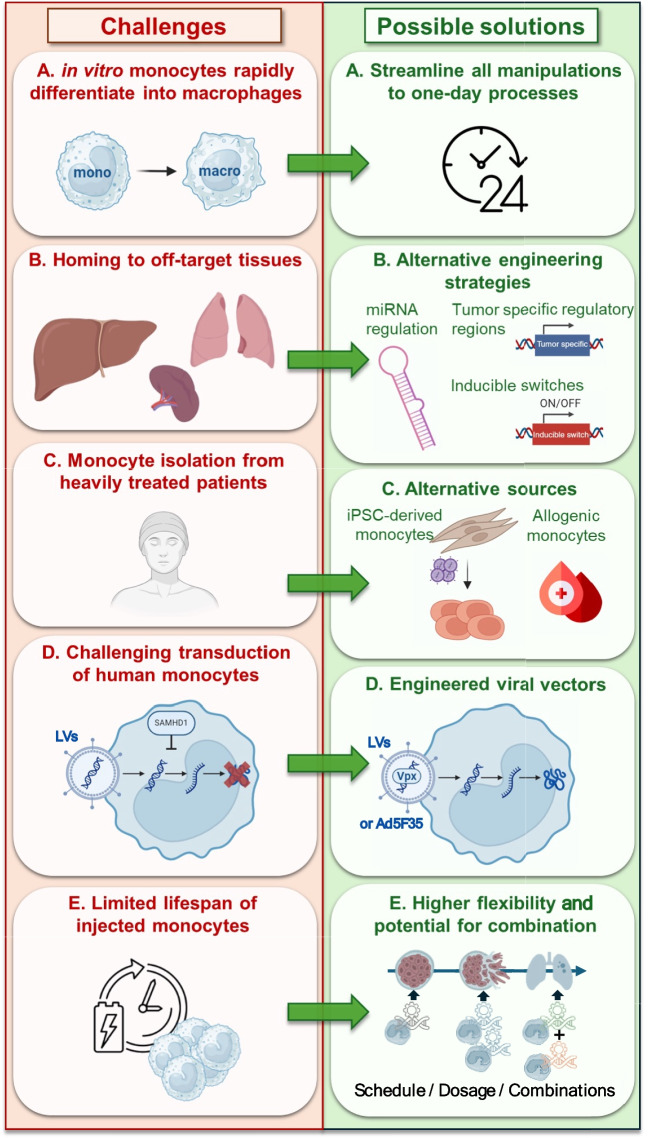
Challenges and possible solution strategies in monocyte-based therapy. **A** Ex vivo cultured monocytes tend to rapidly differentiate into macrophages, reducing the available manipulation times. Therefore, ex vivo handling of monocytes should be streamlined to one-day processes. **B** Injected monocytes might home to off-target organs, such as the spleen, liver and lungs. This can be counteracted by implementing advanced engineering strategies such as: miRNA regulation to inhibit transgene expression in off-target tissues; exploiting tumor specific regulatory region to selectively switch transgene expression only within the tumors; exploring the use of inducible switches. **C** Alternative monocytes sources can be explored to overcome the compromised isolation of monocyte from heavily treated patients. These include iPSC derived-monocytes or monocytes derived from partially or fully HLA-matched donors. **D** To overcome limited lentiviral (LV) transduction efficacy in human monocytes, addition of the Vpx protein into the viral vector production or the use of Ad5f35 can be implemented. **E** The limited lifespan of monocytes may only induce transient therapeutic effects. This offers high flexibility in modulating schedule, dosing and therapeutic combinations according to tumor progression and evolution

When compared with HSC transplantation, because monocytes do not stably engraft, they are expected to provide only a transient effect, which can be seen as both an advantage and a disadvantage. It is though clear that it is easier to provide a booster injection to increase the strength of a transient effect than reversing a chronic and stable change in the entire myeloid lineage. Moreover, transient effects may allow for higher flexibility in the design of the treatment plans as schedules, doses, as well as type and combinations of therapeutic molecules delivered can be adapted as the disease progresses (Fig. 3E). Although monocytes are attractive candidates for adoptive transfer therapies, it is however recognized that these cells need to be handled with particular care to avoid polarization of the derived macrophages towards pro-tumor, immunosuppressive phenotypes. As in the case of macrophages, this can be achieved by engineering the monocytes to direct their differentiation and polarization into macrophages with anti-tumor properties and/or combining with reprogramming treatments. When combined with the delivery of immunomodulatory molecules and/or expression of CARs, adoptive transfer of engineered monocytes might represent a clinically translatable as well as cost- and time-effective cell-therapy approach allowing high degrees of dose and schedule modulation.

#### Shaping the tumor microenvironment: autologous monocytes combined with type I and type II IFNs

The landmark preclinical work from Nakashima and colleagues paved the way for the first in-human clinical trial exploring the use of a monocyte-based cell therapy [88, 89]. The group has developed a cell therapy based on the intraperitoneal administration of autologous monocytes combined with IFNα−2b and IFNγ−1b. In preclinical studies, this combination therapy showed strong synergistic antitumor activity in the OVCAR-3 and LOX xenograft models of ovarian cancer and melanoma, respectively. The demonstrated mechanism of action included enhanced macrophage polarization to an anti-tumor phenotype and consequent macrophage-dependent induction of cancer cell apoptosis.

This was confirmed in a phase I, dose escalation clinical trial (NCT02948426) specifically designed for patients with platinum-resistant/refractory ovarian cancer [89–91]. The trial results showed a clear upregulation of the TRAIL receptor on patients’ monocytes mediating cell-to-cell interaction with cancer cells [89]. This was associated with the activation of the extrinsic apoptotic pathway via caspase-8 in cancer cells. Upregulation of pro-inflammatory cytokines in the peripheral blood of patients was also observed, thus suggesting a rewiring towards an inflammatory TME, which could foster higher CD8^+^ T cell activation. Interestingly, long-term responders showed lower levels of regulatory T cells and lower neutrophil/lymphocyte ratios compared to the baseline. A higher level of MDSCs was also found, suggesting the possible efficacy of combination therapies with agents targeting MDSCs. Overall, the treatment was tolerated with no grade 4 toxicities, whereas the most common grade 3 toxicities were decreased lymphocyte count (33.3%) and abdominal pain (11.1%). There was one grade 3 skin infection, due to a peritoneal catheter that resolved with appropriate antibiotic treatment and removal of the catheter. In terms of clinical outcome, the treatment showed preliminary evidence of antitumor activity. Out of 9 measurable patients, 2 experienced a partial response, 5 resulted in stable disease, and 3 had progressive disease by RECIST criteria [89, 90]. The main limitations of the study were the heavily pretreated patient population with a median of 5 prior treatment regimens and the limited sample size. Despite that, this first-in-human clinical trial exploiting monocytes proved the safety of the therapy and determined the recommended phase II dose and the maximum tolerated dose to be investigated in terms of efficacy in subsequent trials.

#### CAR-monocytes

With the great success of CAR-T cell therapies, many groups are trying to apply a similar approach to other immune cell types, such as NK cells, macrophages and, more recently, also monocytes. CAR-monocytes aim to leverage the functions of monocytes, including their tumor homing potential and ability to differentiate into macrophages, interact with the tumor microenvironment, and, if appropriately engineered, to modulate immune responses. The same CAR constructs developed for macrophages can be used for monocytes with the final aim of promoting and maintaining a pro-inflammatory phenotype in the CAR-monocyte-derived macrophages. CAR-monocytes may provide an interesting alternative to CAR T cells because, unlike T cells, monocytes can better migrate to and accumulate within the tumor due to their natural chemotactic responses to inflammatory signals. Moreover, within the tumor bed, CAR-monocytes can differentiate into macrophages or dendritic cells, making them a suitable vehicle for delivering different CAR-based therapies directly to the tumor microenvironment.

Gabitova and colleagues (Carisma Therapeutics) have developed a CAR-Macrophage platform which is currently tested in a phase I clinical trial (NCT04660929). More recently, they transferred the same CAR platform to monocytes and were able to streamline cell manufacturing into a single day process using a rapid (12 h) adenovirus transduction of human CD14^+^ monocytes [92]. In their preclinical work, the group demonstrated that HER2-targeting CAR-monocytes could: (i) differentiate into CAR-Macrophages with a strong proinflammatory profile when in the presence of HER2^+^ tumor cells, (ii) resist subversion mediated by immunosuppressive myeloid cells, and (iii) specifically phagocytose HER2^+^ cells in vitro. Importantly, CAR-monocytes were effective in the HER2^+^ SKOV3 ovarian cancer model as well as in CD34^+^-humanized NSG mice bearing PANC1-HER2 pancreatic cancers. In both cases, treated mice showed a significantly lower tumor burden and improved overall survival. Based on these promising preclinical results, a phase I clinical trial is ongoing (NCT06254807). The study will evaluate the safety, tolerability, and the manufacturing feasibility of the therapy, and will recruit patients with locally advanced or metastatic HER2-overexpressing solid tumors. Relevant examples of CAR-monocyte based therapies may be also found in contexts other than cancer. In the setting of cardiovascular diseases, Chuang et al. recently developed CAR-monocytes targeting the “don’t eat me” signal mediated by CD47, thus facilitating the elimination of phagocytosis-resistant apoptotic cells after they differentiate into macrophages. High levels of CD47 are also found in tumors. Moreover, they coated the macrophages with nanoparticles for the ROS-induced release of Hydroxypropyl β-cyclodestrin, which can be adapted to the release of any relevant cargo in tumors [93].

As for all CAR-based therapies, ensuring the safety of CAR-monocytes is critical. Off-target effects or excessive immune activation could lead to toxicities such as (CRS or neurotoxicity. Recent results from the NCT04660929 trial [78] reported that HER2-targeting CAR-macrophage therapy was well tolerated with no severe CRS suggesting a safer profile than CAR-T therapy. In terms of mechanism, macrophages have a different cytokine profile than T cells, do not massively proliferate upon activation and kill via phagocytosis and not through extensive cytokine release, all of which support lower probability to induce excessive cytokine storm. Whether CAR-monocytes will be also safe remains to be tested, however, they will eventually differentiate into macrophages and mediate tumor cell killing with the same mechanism. Moreover, adequate control of persistence and activity of CAR-monocytes may be more complex compared to T cells, given that monocytes can differentiate into various cell types in the body. This requires careful design of CAR constructs and, potentially, the incorporation of safety switches or suicide genes. Yang et al. recently provided preclinical proof-of-efficacy data for a novel HER2-CAR THP1 therapy involving the iCasp9 suicide gene. CAR-CD3ζ-CD147-iCasp9 allowed controlled proliferation of CAR-THP1 which also showed robust and specific anti-tumor efficacy and an M1-like phenotype [94]. The differentiation potential of monocytes, while advantageous, also poses challenges in ensuring a consistent product during manufacturing. The cells need to be engineered and expanded in a way that preserves their ability to differentiate appropriately upon infusion.

### Why have monocytes been so often overshadowed?

The early success and broad application of T cell-based therapies have overshadowed the possible exploitation of myeloid cells as possible “living drugs”. However, in recent years, increasing efforts have been put into the development of novel cellular therapies based on myeloid cells, given their interesting characteristics, particularly their natural tumor homing ability [95–97]. Among myeloid cells, monocytes have been initially adumbrated by macrophages or other hematopoietic progenitors and have been relatively understudied. Several factors have contributed to the limited consideration of monocytes as a source of cell-based therapies.

### Challenges of ex vivo manipulation of monocytes

Ex vivo manipulation of monocytes is not an easy task and can present some challenges.

First, ex vivo cultured monocytes tend to rapidly differentiate into macrophages. This reduces the working time window for manipulation and ex vivo handling of monocytes should be streamlined to one-day processes (Fig. 3A).

Second, injected monocytes might accumulate in off-target organs, such as spleen, liver and lungs resulting in unwanted side effects. Advanced engineering strategies such as the addition of tumor-specific gene regulatory sequences can overcome this issue (Fig. 3B). This was elegantly achieved by exploitation of either the Tie2 or the MMP-14 promoters as ‘tumor-selective’ switch in macrophages derived from genetically engineered and transplanted HSCs [36–40, 42]. Another interesting method is the introduction of endogenous microRNA–miRNA regulation sequences in the viral vector system to stop transgene expression in off-target cells [98]. Alternatively, inducible switch systems could also be explored [99].

Third, monocyte isolation from heavily treated patients might also be challenging. The use of fully or partially HLA-matched donors could be explored to overcome this limitation. In this context, host cells could potentially react against the injected donor monocytes, but no GvHD should occur, as T cells are the major players of this adverse effect [100]. Moreover, compared to T cells, monocytes have a limited capacity for in vitro expansion. This implies that a higher initial volume of blood or bone marrow is required to obtain a therapeutic dose of these cells, which can be a logistical challenge [95]. Alternatively, iPSCs could be used to create an off-the-shelf bank to be readily differentiated into engineered monocytes upon need [101] (Fig. 3C). Moreover, like macrophages, monocytes do not induce GvHD, therefore, allogeneic monocytes from healthy donors or generated from allogeneic iPSCs could be explored for off-the-shelf therapies. Human monocytes can be difficult to transduce, considering that cells in the G_0_ phase, such as freshly isolated PBMCs, are often resistant to gene transfer strategies [102]. Moreover, myeloid cells are generally refractory to lentiviral infection due to SAMHD1, a myeloid-specific restriction factor blocking early reverse transcription [103]. Recent findings have shown that the viral protein Vpx can counteract this cellular restriction thus paving the way for alternative strategies to circumvent this cellular block [103–105] (Fig. 3D). The chimeric adenoviral vector Ad5F35 is the only viral vector currently used for both macrophage and monocyte engineering in phase I clinical trials (NCT06254807 and NCT04660929). Alternatively, the use of monocytes derived from easy-to-transduce iPSCs, could also be explored [101]. Finally, compared to T cells, monocytes have a limited capacity for in vitro expansion. This implies that a higher initial volume of blood or bone marrow is required to obtain a therapeutic dose of these cells, which can be a logistical challenge [95].

### In vivo challenges

Despite being less heterogeneous than macrophages, monocytes include subsets playing distinct roles in inflammation, immune regulation, and tissue repair [106]. In addition, after entering the tumor tissue, monocytes can differentiate into macrophages or dendritic cells [107]. While this may be advantageous for cancer immunotherapy, a reliable and consistent exploitation of this property for therapeutic purposes seems difficult to achieve. Moreover, monocytes are highly responsive to the tumor microenvironment, resulting in variable differentiation and functional shifts, which need to be appropriately controlled [108]. As in the case of macrophages and neutrophils, ex vivo engineering and/or combination with immunomodulatory treatments will need to be further explored to direct polarization of the injected monocytes. Actual persistence of monocyte-derived macrophages within the tumor needs to be carefully to be investigated. It is, however, expected to be limited and, therefore, the effect of a monocyte-based therapy will likely be transient and require repeated administrations. In perspective, strategies able to engage endogenous adaptive immune responses could at least in part obviate this limitation and lead to durable tumor protection. Moreover, as mentioned above, a transient persistence may allow a more flexible design of the therapeutic strategy (Fig. 3E).

## Conclusions

Preclinical and clinical evidence clearly indicates that the efficacy of T and NK cell-based therapies is limited by the reduced trafficking of infused effectors to solid tumors and by the presence of a strongly immunosuppressive TME. Myeloid cell-based therapies may offer valid alternatives thanks to their inherent functional properties. Indeed, myeloid cells are more abundant than T and NK cells, have a superior ability to home and infiltrate tumors, are associated with reduced toxicity, and can be reprogrammed to have broad effects on the TME being able to limit local immunosuppression and promote the recruitment and activation of other immune cells. Many efforts are currently being made in the field to identify which cell type of the myeloid compartment is the more suitable to develop safe, effective and easy to manufacture cell therapies. Macrophage-based therapies and, particularly CAR-Macrophages, are emerging as a more advanced and potentially effective alternative to T and NK cell-based therapies, with improved engineering solutions playing a critical role in their development. The clinical applicability of macrophage-based therapies, however, is still limited by manufacturing complexity and by their in vivo plasticity, which require mechanism-based therapeutic combinations to prevent their functional polarization towards pro-tumor phenotypes. The lesson we are learning from macrophage-based therapies can now be extended to other myeloid cells such as monocytes, whose therapeutic potential has been recently regained interest in the field. Available data clearly indicate that, compared to macrophages, monocytes have the advantage of being easier to isolate and do not require expensive and time-consuming in vitro differentiation steps. Importantly, monocytes show a more efficient trafficking and homing to tumors than macrophages, thus constituting a potentially preferred cell vehicle to target solid tumors. These properties are allowing a rapid expansion and improvement of monocyte-based therapies, whose safety and efficacy will be the objective of a progressively higher number of clinical trials involving patients with solid tumors.

## Data Availability

No datasets were generated or analysed during the current study.

## Abbreviations

CAR: Chimeric antigen receptor
CAR-M: Chimeric antigen receptor - macrophages
CAR-Ps: Chimeric Antigen Receptors for Phagocytosis
CCL: C-C motif chemokine ligand
CCR: C-C motif chemokine receptor
CRISPR-Cas9: Clustered Regularly Interspaced Short Palindromic Repeats-CRISPR-associated 9
CRS: Cytokine release syndrome
CSF: Cerebrospinal fluid
ECM: Extracellular matrix
FcR: Fc receptor γ-chain
GBM: Glioblastoma
GMP: Good manufacturing practice
GvHD: graft versus host disease
HER2: human epidermal growth factor receptor 2
HSC: Human epidermal growth factor receptor 2
ICANS: Immune effector cell-associated neurotoxicity syndrome
IFN: Interferon
IL: Interleukin
iNKT cells: Invariant natural killer T cells
iPSCs: induced pluripotent stem cells (iPSC)
ISGs: Interferon Stimulated Genes
ITAM: Immunoreceptor tyrosine-based activation motif
MAIT cells: Mucosal-associated invariant T cells
MDSCs: Myeloid-derived suppressor cells
MMP9: Matrix metalloproteinase-9
Megf10: Multiple EGF-like-domains protein 10
NK cells: Natural killer cells
OTOT: On-target off-tumor
PD-1: Programmed cell death protein 1
PD-L1: Programmed cell death protein 1 - ligand
PI3K: Phosphoinositide 3-kinase
scFv: Single chain antibody fragment
TAMs: Tumor-associated macrophages
Treg cells: Regulatory T cells
TGF-β: Transforming growth factor beta
TME: Tumor microenvironment
TAMs: Tumor-associated macrophages
TILs: Tumor infiltrating lymphocytes
